# Minimal Change Disease and IgA Deposition: Separate Entities or Common Pathophysiology?

**DOI:** 10.1155/2013/268401

**Published:** 2013-05-21

**Authors:** Brandon S. Oberweis, Aditya Mattoo, Ming Wu, David S. Goldfarb

**Affiliations:** ^1^Department of Medicine, New York University, School of Medicine, New York, NY, USA; ^2^Division of Nephrology, New York University, School of Medicine, New York, NY, USA; ^3^Department of Pathology, New York University, School of Medicine, New York, NY, USA; ^4^Nephrology Section, New York Harbor VA Medical Center, 111G New York DVAMC, 423 E. 23 Streat New York, NY 10010, USA

## Abstract

*Introduction*. Minimal Change Disease (MCD) is the most common cause of nephrotic syndrome in children, while IgA nephropathy is the most common cause of glomerulonephritis worldwide. MCD is responsive to glucocorticoids, while the role of steroids in IgA nephropathy remains unclear. We describe a case of two distinct clinical and pathological findings, raising the question of whether MCD and IgA nephropathy are separate entities or if there is a common pathophysiology. *Case Report*. A 19-year old man with no medical history presented to the Emergency Department with a 20-day history of anasarca and frothy urine, BUN 68 mg/dL, Cr 2.3 mg/dL, urinalysis 3+ RBCs, 3+ protein, and urine protein : creatinine ratio 6.4. Renal biopsy revealed hypertrophic podocytes on light microscopy, podocyte foot process effacement on electron microscopy, and immunofluorescent mesangial staining for IgA. The patient was started on prednisone and exhibited dramatic improvement. *Discussion*. MCD typically has an overwhelming improvement with glucocorticoids, while the resolution of IgA nephropathy is rare. Our patient presented with MCD with the uncharacteristic finding of hematuria. Given the improvement with glucocorticoids, we raise the question of whether there is a shared pathophysiologic component of these two distinct clinical diseases that represents a clinical variant.

## 1. Introduction

Minimal Change Disease (MCD) is the most common cause of nephrotic syndrome in children, while IgA nephropathy is the most common cause of glomerulonephritis worldwide. MCD is responsive to glucocorticoids, while the role of steroids in IgA nephropathy remains unclear. We describe a case of two distinct clinical and pathological findings, raising the question of whether MCD and IgA nephropathy are separate entities or if there is a common pathophysiology.

Minimal Change Disease (MCD) is the most common cause of nephrotic syndrome in children. It accounts for 70–90% of cases in children less than 10 years of age and 50% of cases in older children [1]. Clinical symptoms are typically preceded by an upper respiratory infection or recent use of medication. Common clinical manifestations include abrupt onset of edema, proteinuria, hyperlipidemia, and hypoalbuminemia [2]. 

IgA nephropathy is the most common cause of glomerulonephritis worldwide. The prevalence of IgA nephropathy is significantly higher in Asia than in the United States. IgA nephropathy is present in 40% of biopsies for glomerular disease in Asia, compared to 10% of biopsies in the United States. IgA nephropathy most commonly presents with painless, gross hematuria or microscopic hematuria, and mild proteinuria, within days of pharyngitis, tonsillitis, or, less commonly, gastroenteritis [3].

MCD is well known to be responsive to glucocorticoids, while the role of steroids in IgA nephropathy remains unclear. Previous studies on coexisting MCD and IgA deposition have shown that, with the administration of steroids, subsequent biopsies reveal the resolution of IgA deposition, a finding that does not occur in IgA nephropathy alone. Therefore, the question arises of whether MCD and IgA nephropathy are distinctly separate entities or if there is a component of shared pathophysiology.

## 2. Case Report 

A 19-year-old Chinese man with no significant past medical history presented to the Emergency Department with a 20-day history of anasarca, frothy urine, and multiple pruritic papules on the extensor surfaces of his upper extremities. He also noted a recent decrease in urine output and diffuse muscle aches. Physical examination was notable for blood pressure of 144/85 mmHg, a liver edge palpable 2 cm below the costal margin, 3+ pitting edema in his face, upper and lower extremities, erythematous papules on the extensor surfaces of his upper extremities bilaterally, and Terry's nails. The remainder of the physical examination was unremarkable. 

On admission, his basic metabolic panel revealed the following: Na 128 mEq/L, K 5.2 mEq/L, BUN 68 mg/dL, and Cr 2.3 mg/dL. A urinalysis was significant for dark yellow urine, 3+ RBCs, 3+ protein, 5–10 WBCs, 5–10 RBCs, and fine granular mixed cells. His urine protein : creatinine ratio was 6.4, and his estimated glomerular filtration rate (eGFR) was 36 mL/min/1.73 m^2^. AST and ALT were 151 U/L and 108 U/L, respectively. The elevation in transaminases prompted determination of hepatitis serologies, which yielded positive hepatitis B surface antigen (HBsAg), anti-HBc IgM, and HBeAg. His hepatitis B viral load was found to be >1.10 × 10^8^ copies/mL. Serologic studies included negative anti-dsDNA antibody and ANA and normal C3 (85.5) and C4 (28.6).

**Figure 1:**
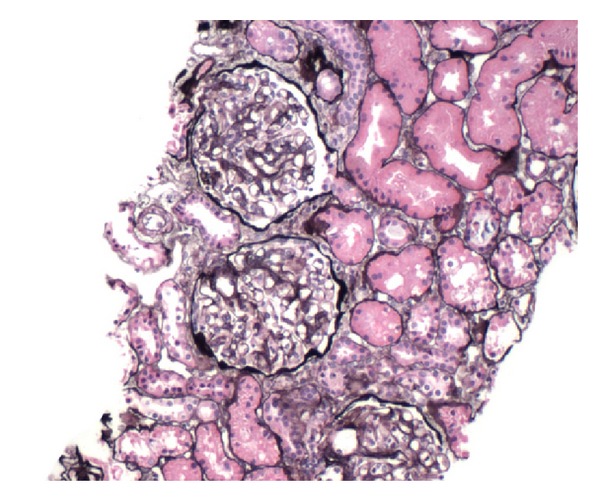
Light microscopy of renal biopsy with Jones' silver stain.

**Figure 2:**
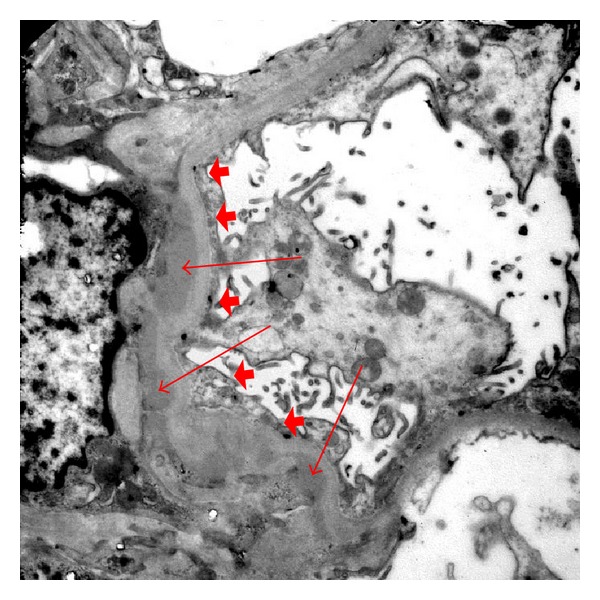
Electron microscopy of renal biopsy with paramesangial deposits (arrows) and foot process effacement (arrow heads).

A percutaneous renal biopsy was performed. Light microscopy with Jones' silver stain revealed hypertrophic podocytes, patchy interstitial fibrosis, and tubular atrophy of 5% of the tubulointerstitial compartment (Figure 1). Electron microscopic evaluation revealed mesangial areas of normal cellularity with numerous electron dense deposits in mesangial and paramesangial locations (Figure 2). The podocytes had extensive foot process effacement. Immunofluorescence staining showed the mesangium is stained strongly positive for IgA, C3, and kappa and lambda light chains. The clinical history, podocyte effacement, and immunofluorescence were considered consistent with the diagnosis of MCD with IgA deposition. 

The patient was immediately started on entecavir 0.5 mg every 2 days for his active hepatitis B infection. Following a negative PPD, prednisone 60 mg daily was initiated for treatment of his MCD.

The patient exhibited dramatic improvement in his frothy urine and anasarca within the first 2 weeks of therapy. Repeat urinalysis showed no hematuria or proteinuria, urine protein : creatinine ratio was 0.5, and basic metabolic panel showed a return in creatinine to baseline (Table 1). The prednisone 60 mg daily was continued for 8 weeks and was then tapered by 5 mg every 3 days. On the last day of the prednisone taper, the patient's physical examination was notable for bilateral upper and lower extremity edema, as well as facial edema. Given the relapse of nephrotic syndrome, the patient was restarted on high-dose steroids, and a longer steroid taper was implemented following clinical remission. However, upon subsequent taper, the patient had recurrent nephrotic range proteinuria and hematuria, indicating that he had steroid-dependent MCD. The patient chose steroid sparing management with mycophenolate mofetil (MMF) for toxicity reasons, and a slow steroid taper has again begun. Under this regimen the patient again had remission of the nephrotic syndrome and has no hematuria on urinalysis but still remains on a minimal amount of alternate day prednisone. 

## 3. Discussion 

Characteristic cases of MCD have an overwhelming improvement in clinical symptoms with the administration of steroids [2]. Conversely, fewer than 10% of patients with IgA nephropathy have resolution of urinary manifestations with the administration of steroids [4]. Given the evidence that IgA mesangial deposits resolve with the use of steroids in the setting of MCD, these two syndromes may be different clinical manifestations sharing a common pathophysiologic component [5–7]. 

The underlying etiology of MCD has not yet been elucidated. However, the pathogenesis is hypothesized to occur through activation of CD4+ and CD8+ T cells, as well as subsequent activation of NF*κ*B and IL-1, IL-6, IL-2, IL-8, and TNF-*α*. These cytokine and inflammatory mediators are thought to damage the glomerular foot processes, leading to proteinuria and other sequelae of nephrotic syndrome [8]. Similarly, several studies have shown the immediate recurrence of MCD following kidney transplantation. These findings advocate the role of a humoral factor causing increased glomerular permeability [9]. 

Subsequent studies have hypothesized that there is a glomerular permeability factor contributing to the findings of nephrotic syndrome in MCD. The factor is possibly a lymphokine produced by T cells [10]. Furthermore, recent studies have found that T cell deposition in renal tubules in IgA nephropathy may be a marker of progression and severity [11]. This finding implicates a role of T cell activation and dysfunction in underlying IgA nephropathy. Although these findings do not necessitate a relationship between MCD and IgA nephropathy, the role of T cell dysfunction is possibly an underlying relationship between the two processes.

Our case highlights two distinct pathological findings. Findings consistent with MCD are minimal changes on light microscopy with electron microscopy demonstrating foot process effacement. IgA nephropathy findings include electron dense mesangial deposits that stain positive for IgA. Clinically, however, our patient presented with minimal change in nephrotic syndrome with the uncharacteristic findings of hematuria, which can be attributed to IgA deposition. We postulate that there may be a clinical variant of MCD, which has the pathological findings of IgA deposition. 

Furthermore, the evidence in the literature of resolution of IgA deposition with steroids, in the setting of MCD, further supports this hypothesis [5–7]. It is important to note that our patient was found to have a diagnosis of hepatitis B. Some research suggests that glomerular IgA deposition may occur in the setting of liver disease. The pathophysiology includes impaired IgA clearance by lysosomal degradation in hepatocytes via asialoglycoprotein receptors, as well as Fc receptors for IgA on Kupffer cells [8]. However, if impaired hepatic clearance was the primary pathologic mechanism, there would not be significant improvement in IgA deposition with the administration of steroids. Furthermore, the findings of urinalysis that the patient's hematuria correlated with lower extremity edema and nephrotic range proteinuria supports the hypothesis that MCD with IgA deposition represents a clinical variant of MCD.

## Figures and Tables

**Table 1:** Laboratory data and clinic symptoms.

Date	Urine RBC	Urine protein	Clinical symptoms	Treatment
Sep. 2009	3+ (5–10 RBC/hpf)	3+	Edema	Time of diagnosis before therapy
Jan. 2010	0	0	No symptoms	Steroids
Mar. 2010	3+ (5–10 RBC/hpf)	3+	Edema	Steroids tapered off 1 week prior
Oct. 2010	2+ (5–10 RBC/hpf)	3+	Edema	MMF and low dose steroids
Dec. 2010	0	0	No symptoms	MMF and low dose steroids
